# Correlation analysis between the complex electrical permittivity and relaxation time of tissue mimicking phantoms in 7 T MRI

**DOI:** 10.1038/s41598-022-19832-y

**Published:** 2022-09-14

**Authors:** Daniel Hernandez, Kyoung-Nam Kim

**Affiliations:** 1 Department of Biomedical Engineering, Gachon University, Incheon, Republic of Korea; 2 Department of Health Sciences and Technology, Gachon University, Incheon, Republic of Korea

**Keywords:** Biomedical engineering, Electrical and electronic engineering

## Abstract

Dielectric relaxation theory describes the complex permittivity of a material in an alternating field; in particular, Debye theory relates the time it takes for an applied field to achieve the maximum polarization and the electrical properties of the material. Although, Debye’s equations were proposed for electrical polarization, in this study, we investigate the correlation between the magnetic longitudinal relaxation time T1 and the complex electrical permittivity of tissue-mimicking phantoms using a 7 T magnetic resonance scanner. We created phantoms that mimicked several human tissues with specific electrical properties. The electrical properties of the phantoms were measured using bench-test equipment. T1 values were acquired from phantoms using MRI. The measured values were fitted with functions based on dielectric estimations, using relaxation times of electrical polarization, and the mixture theory for dielectrics. The results show that, T1 and the real permittivity are correlated; therefore, the correlation can be approximated with a rational function in the case of water-based phantoms. The correlation between index loss and T1 was determined using a fitting function based on the Debye equation and mixture theory equation, in which the fraction of the materials was taken into account. This phantom study and analysis provide an insight into the application relaxation times used for estimating dielectric properties. Currently, the measurement of electrical properties based on dielectric relaxation theory is based on an antenna, sometimes invasive, that irradiates an electric field into a small sample; thus, it is not possible to create a map of electrical properties for a complex structure such as the human body. This study could be further used to compute the electrical properties maps of tissues by scanning images and measuring T1 maps.

## Introduction

Magnetic resonance imaging (MRI) can be used to acquire the electrical properties of tissues. The electrical properties, especially conductivity, can be used as a biomarker to identify possible cancerous tumors^1–4^. Electrical property tomography (EPT) can be used to determine the electrical properties^5,6^_._ It has attracted attention in several fields^7–10^. The use of EPT has been proposed to compute the electrical permittivity and conductivity based on the Helmholtz equation. The manipulation of the Helmholtz equation leads to a representation of the conductivity and permittivity in relation to the Laplacian of the magnetic field |B1|^11,12^. This method requires a condition in which the gradient of the sum of permittivity and conductivity is zero. The real and imaginary parts of |B1| are used to compute the permittivity and conductivity, respectively, and the conductivity can be simplified using the phase of |B1|. One drawback of this method is the application of the Laplacian, as taking a double derivative leads to an increase in noise. Therefore, several methods for de-noising^13–15^, filtering^16^, and estimating the derivative^17^ have been proposed. Particularly, permittivity mapping using this method leads to strong artifacts, and limited research has been conducted based on the use of permittivity. This method of using the Laplacian of the |B1|-field is the standard for EPT, with applications in both low- and high-frequency imaging^18,19^, cancer analysis^1^, and animal imaging^20^.

Another method has been proposed^14,21^ to address the limitations of the Laplacian method. In this method, the Maxwell mixture theory, and the correlation between the water content of tissues and their electrical properties are used. This method has the advantage of conserving image quality. Water-based EPT (wEPT) is based on correlating the electrical properties of the tissues and the water content, where the water content is estimated using T1-weighted images. It proposes the use of a curve-fitting equation based on measured data for the estimation of permittivity and conductivity. However, this method does not consider the intracellular conductivity interactions of Na + and has limited applicability in experiments^22^. It should be noted that the existing studies used a limited number of samples, and the model was applied for water concentrations higher than 65%. Following the water content concept, another method for computing the EPT was presented^23^, in which the diffusion tensor (DT) was used as a metric to compute conductivity. The disadvantages of DT are that it is prone to artifacts and low image resolution.

Dielectric relaxation measures the electrical properties of a single material. It requires a sample of the material and a dedicated antenna for transmission and reception of the electric field at different frequencies. This theory describes that the permittivity and conductivity are related to the frequency and relaxation time. This relaxation time is the time required for a material to reach the maximum polarization when an electric field is applied. This method can be used for analyzing single materials and not for complex structures such as the human body. In magnetic resonance, there are also relaxation times; in particular, the longitudinal relaxation, also known as T1, which indicates the time that the magnetization vector requires to recover to the equilibrium state along the B_0_ field after the application of a radiofrequency field. Notably, the times described by dielectric relaxation theory and T1 relaxation time are different. However, if there is a correlation between the T1 and electrical properties, MRI could be used to estimate the permittivity and conductivity of tissues in vivo and during clinical procedure.

For the previous reasons, the goal of this study is to investigate the correlation between the longitudinal relaxation time T1 and the complex permittivity. The understanding of this relationship can help perform further research on the application of permittivity in clinical use. Furthermore, as the imaginary part of the permittivity is related to the conductivity, it can be used as another tool to measure the conductivity of tissues. Finding the relationship between T1 and complex permittivity can improve the wEPT method, in which a more direct computation can be done, without first estimating the water content, while at the same time keeping the high resolution of the image. This correlation also would remove the limitation of the wEPT that was modeled for tissues with more than 60% of water content.

## Theory

### T1 mapping

The inversion recovery pulse sequence is the standard sequence for computing T1 maps despite requiring a long acquisition time. T1 is computed by solving the relaxation equation.

1 $$S = \rho \left( {1 - 2e^{{ - \frac{TI}{{T1}}}} + e^{{ - \frac{TR}{{T1}}}} } \right)$$

where S is the acquired MRI signal, ρ is the proton density, TI is the inversion time, TR is the repetition time, and T1 is the longitudinal relaxation time. A nonlinear least-squares fitting method is used to estimate the value of T1. The use of inversion recovery for T1 mapping can introduce errors due to the nonuniformity of B1.

Another method to compute T1 maps is by using magnetization-prepared rapid gradient echo (MPRAGE), which is based on a series of gradient echoes with an inversion time TI, using a nonselective inversion pulse of 180°, which requires short TE and small flip angles. The reconstruction of the T1 map is based on previously reported work^24^. This method requires three images, each with different TI values, low, medium, and long, as the other parameters are constant. For the reconstruction the follow equation for the multichannel RF coil is used:

2 $$\frac{{\mathop \sum \nolimits_{k} \left( {\left\| {S_{{TImax}}^{k} } \right\| - \frac{{S_{{TImin}}^{k} \left\| {S_{{TImax}}^{k} } \right\|}}{{S_{{TImax}}^{k} }}} \right)}}{{\mathop \sum \nolimits_{k} \left( {\left\| {S_{{TImax}}^{k} } \right\| - \frac{{S_{{TImed}}^{k} \left\| {S_{{TImax}}^{k} } \right\|}}{{S_{{TImax}}^{k} }}} \right)}} = \frac{{e^{{\frac{{TImax - TImin}}{{T1}}}} - 1}}{{e^{{\frac{{TImax - TImed}}{{T1}}}} - 1}}$$

where k is the channel number, and TImax, TImed, and TImin are the inversion times for the long, medium, and short channels, respectively. STImax, STImed, and STImin are the images acquired with long, medium, and short TI, respectively. T1 is the value for which this equation must be solved. The solution to this equation requires the use of a look-up table. On the left side of the equation, the values of TImax, TImed, and TImin are known. By varying the value of T1 from low to high, the lookup table compares the values between the right and left sides of the equation. This method is faster than the inversion recovery method that requires curve fitting.

### Water content based EPT

In the previous work^14^, a method to compute electrical properties based on the water content was proposed, in which a relationship between T1 and water content was established as

3 $$W = \frac{1}{A + B/T1}$$

where A and B are coefficients for the field strength, the water maps were then used to estimate the conductivity and permittivity with the fitting functions

4.1 $$\sigma = c1 + c2e^{c3W}$$

4.2 $$\varepsilon = p1W^{2} + p1W + p3$$

where W is the water content measured from the T1, and the coefficients, c1, c2, c3, p1, p2 and p3 were found by regression fitting.

### Dielectric relaxation theory

To fit the data between the measured T1 and permittivity, we used a rational function that resembles the structure of the equations of dielectric relaxation and mixture theories. The use of relaxation times to compute the permittivity and conductivity, also known as dielectric relaxation^25^, is best described by the Debye equations. Debye equations are derived from the electrical polarization of electrons and relate the time required for alignment with the applied field^26,27^. The Debye equations represent the complex permittivity ε*, as follows:

5 $$\varepsilon^{*} = \varepsilon^{\prime} + j\varepsilon^{\prime\prime}$$

where the real part ε’ is the electrical permittivity, and the imaginary part ε’’ is commonly referred to as the loss index, which is related to the electrical conductivity as follows:

6 $$\sigma = w\varepsilon_{0} \varepsilon ^{\prime\prime}$$

where *w* is the frequency and *ε*_0_ is the permittivity of free space. These equations are considered to be applicable to polar dielectrics^26^. With these definitions, the Debye equations can be used to describe the permittivity as follows

7.1 $$\varepsilon^{\prime} = \varepsilon_{\infty } + \frac{{\varepsilon_{s} - \varepsilon_{\infty } }}{{1 + \left( {wt} \right)^{2} }}$$

7.2 $$\varepsilon^{\prime\prime} = \frac{{(\varepsilon_{s} - \varepsilon_{\infty } )wt}}{{1 + \left( {wt} \right)^{2} }}$$

where *w* represents the frequency, *t* is the electrical polarization relaxation time, *ε*_s_ is the permittivity at zero frequency, and *ε*_∞_ is the permittivity at a high frequency. For water, ε_s_ and ε_∞_ are approximately 80 and 45^28^, respectively. The same report shows that for a sample mixed with water and methanol at 25 °C, *ε*’ is approximately 80 and 30 when the concentration of water is 100% and 0%, respectively, when the frequency is 300 MHz.

Although Eq. 7 was developed for the relation of the electric field, we only use it as a reference for curve fitting following the structure of the rational equation. Given the complex representation of the permittivity, it can also be expressed in a complex diagram, a concept upon which the Cole–Cole diagram is based. It can be expressed as follows.

8 $$\left( {\varepsilon^{^{\prime}} - \frac{{\varepsilon_{s} + \varepsilon_{\infty } }}{2}} \right)^{2} + \left( {\varepsilon^{\prime\prime}} \right)^{2} = \left( {\frac{{\varepsilon_{s} - \varepsilon_{\infty } }}{2}} \right)^{2}$$

This equation also resembles a circle with a center at [(ε_s_ + ε_∞_)/2, 0] and a radius of (ε_s_ − ε_∞_)/2. If the measured points fall into the semicircle, the material is considered to exhibit a Debye relaxation. There have been other equations and relationships for materials that do not follow the Debye-type relaxation. The Fouss-Kirkwood equation is a generalization for most materials and is given as follows:

9 $$\varepsilon^{\prime\prime} = \varepsilon_{\infty } + \left( {\varepsilon_{s} - \varepsilon_{\infty } } \right)\delta \frac{{\left( {\omega \tau } \right)^{\delta } }}{{1 + \left( {\omega \tau } \right)^{2\delta } }}$$

Parameter δ is a value between 0 and 1 and is related to the type of material. This equation is similar to Eq. 7.2, when δ = 1.

The mixing theory has also been proposed to analyze the effective permittivity when two or more materials are combined, given certain material properties^29^. The general formulation of this theory is given by the Maxwell Garnet formula, which estimates the effective permittivity *ε*_*eff*_ of a material composed of a background material with permittivity *ε*_*e*_ and an inserted material of permittivity *ε*_*i*_*.* The formula is given as follows.

10 $$\frac{{\varepsilon_{eff} - \varepsilon_{e} }}{{\varepsilon_{eff} + 2\varepsilon_{e} }} = f\frac{{\varepsilon_{i} - \varepsilon_{e} }}{{\varepsilon_{i} + 2\varepsilon_{e} }}$$

The relationship between the materials is given by the volume fraction *f* of the inserted material. *ε*_*eff*_ represents the complex permittivity, for which the conductivity can be estimated for a small *f* as follows:

11 $$\sigma_{eff} \approx \frac{{9\varepsilon_{e}^{2} f\sigma_{i} }}{{\left( {\varepsilon_{i} + 2\varepsilon_{e} } \right)^{2} + \frac{{\sigma_{i}^{2} }}{{\omega^{2} }}}}$$

where the conductivity of the inclusion is given by *σ*_*i*_.

## Methods

### Phantom construction

**Figure 1:**
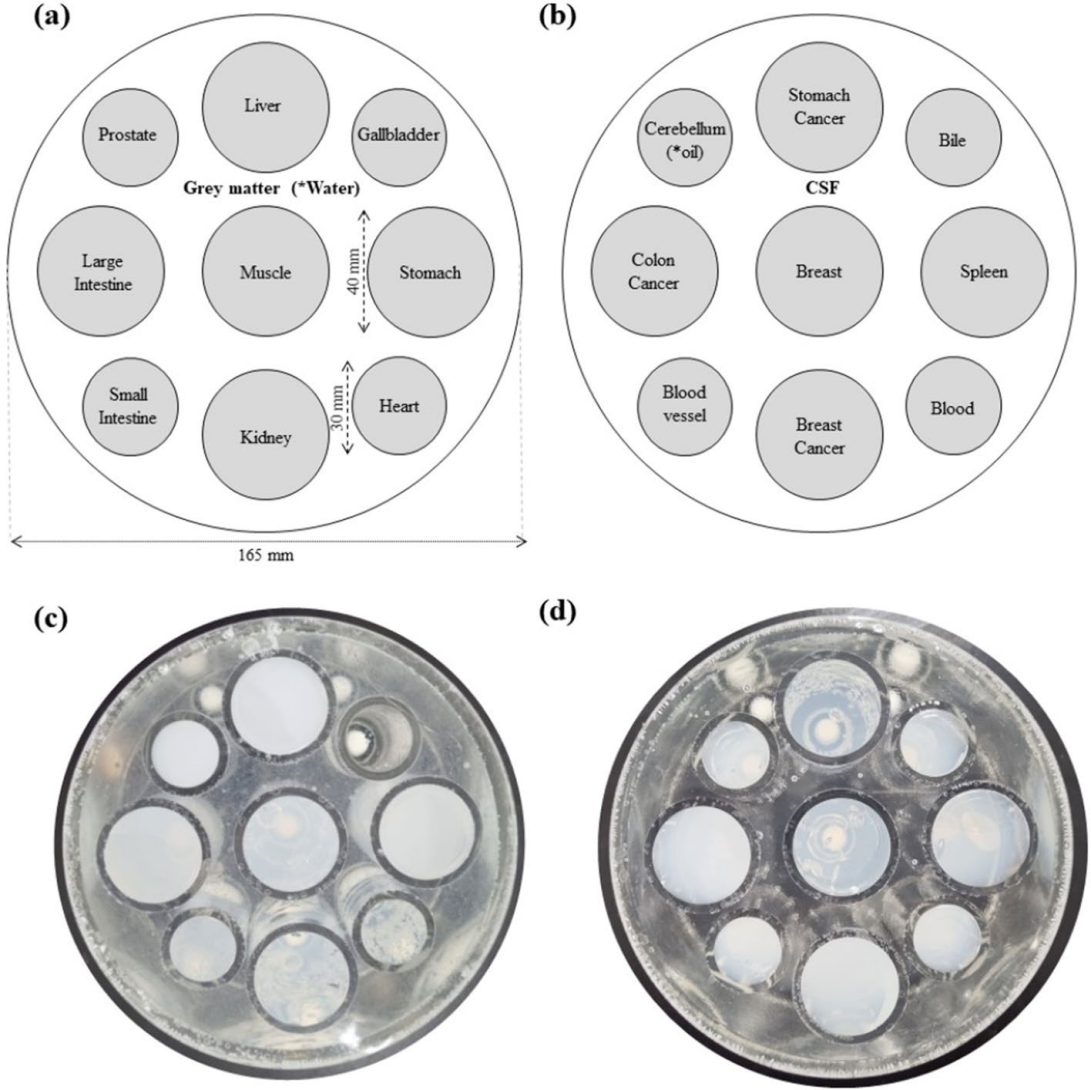
The design of the two phantoms used and the localization of each of the mimicking tissues. The phantoms are labeled as (**a**) phantom 1, and (**b**) phantom 2. The elements with asterixis (water and oil) indicate that they were included in the MPRAGE image and were switched to grey matter and cerebellum for IR images. The pictures of the manufactured (**c**) phantom 1 and (**d**) phantom 2, which were used for IR imaging.

Two phantoms were developed for this study. Each phantom had ten samples, with each sample having different electrical properties. The samples were cylinders with radii of 82.5 mm and heights of 150 mm, as illustrated in Fig. 1. The samples inside the phantom had two different sizes, with large and small cylinders having radii of 20 mm and 15 mm, respectively. To adjust the electrical properties, we used a procedure from previous research^30^, which consist on mixing water, sodium chloride (NaCl), sucrose, and agar. Each of the samples inside the phantom represents one type of human tissue, and we selected a variety of tissues from the brain, upper body, lower body, and cancer-mimicking tissues, as described in Table 1. The percentages of the three main ingredients (water, NaCl, and sucrose, with exception of agarose) for construction of the phantoms are summarized in Table 1. The electrical properties of these tissues were selected from the Sim4life (Zurich MedTech AG) database, which is based on Gabriel’s previous work^31^, and from the data of the colon, breast, and stomach cancers^2,32,33^. An online calculator (https://amri.ninds.nih.gov/cgi-bin/phantomrecipe) and MATLAB code provided by the authors were used to match the electrical properties. The location of each tissue type for each phantom is illustrated in Fig. 1a,b for phantom 1 and 2, respectively. Pictures of the phantoms used for IR imaging are displayed in Fig. 1c,d for phantom 1 and 2, respectively.

**Table 1:** The phantom composition and the measured complex permittivity for each tissue-mimicking sample.

Tissue type	Water [%]	NaCl [%]	Sugar [%]	ε’	ε’’
Gray matter (brain)	50.76	1.48	46.90	60.22	41.43
Muscle	50.48	1.72	47.04	59.65	50.97
Prostate	57.41	1.64	40.09	64.16	63.15
Stomach	66.69	1.12	31.19	68.47	60.61
Gallbladder	56.40	1.96	40.80	63.41	71.97
Heart	67.98	0.99	30.01	68.98	56.47
Kidney	71.73	0.99	26.21	70.30	61.85
Large intestine	58.50	1.25	39.37	64.91	51.72
Small intestine	69.12	2.03	27.82	68.78	110.89
Liver	45.54	1.74	52.03	55.51	40.19
CSF	78.65	1.89	18.28	71.82	128.46
Bile	83.22	1.24	14.29	73.87	96.28
Blood	60.60	1.95	36.54	65.56	81.31
Blood vessel	41.19	1.92	56.26	51.27	32.94
Breast gland	55.07	1.53	42.57	62.88	52.81
Spleen	62.64	1.29	35.12	66.81	59.54
Cerebellum	50.96	2.21	46.06	59.70	58.38
Cancer stomach	84.88	0.74	13.10	74.85	64.46
Cancer colon	65.44	1.46	32.11	67.86	73.09
Cancer breast	48.89	1.68	48.70	58.43	45.43
Water	100	0	0	78.64	11.69
Oil [100%]	0	0	0	11.7	0.45

### Dielectric measurement

To verify the electrical properties of the constructed phantoms, we used a dielectric assistance kit (DAK model 12, SPEAG, Zurich, Switzerland), and the data were collected for each phantom sample with a frequency bandwidth range of 200–400 MHz. We collected the real and imaginary permittivity components or loss index values for each sample. The values are listed in Table 1. During the preparation of the phantoms, one portion of the mixture was used to fill the phantom for imaging (Fig. 1) and another portion was used to fill a cylindrical container of 400 ml for each individual mixture. These containers were used for measuring the dielectric properties by placing the DAK probe in direct contact. Measurements were performed with each individual mixture inside of the container, this set up was surrounded by open air, to reduce interaction with other objects.

### MRI experiments

To create T1 maps, we performed experiments with a 7 T MRI scanner (Siemens, Magnetom, Siemens Healthcare, Erlangen, Germany) using a conventional turbo inversion recovery and MPRAGE sequence, for which images were acquired with different inversion times TI with a large TR value constant.

To compute the T1 values, we acquired turbo inversion recovery images with five different TI values of 200, 500, 1000, 2000, and 4000 ms, while the TR was set to 4160 ms and the FA was 120°. T1 values were fitted using the method described in Eq. (1).

The MPRAGE pulse sequence was acquired with a TR of 2200 ms, a TE of 4 ms, and an FA of 9°. Three images were acquired with inversion times TI of TImax 1600 ms, TImed 480 ms, and TImin 60 ms. We used a built-in 8-channel TRx coil for imaging. For the computation of T1, we used a look-up table that consisted of T1 values ranging from 50 to 4500 ms with a step size of 0.1 ms.

### Data analysis of real permittivity and T1

Using the measured electrical properties and computed relaxation times T1, we fitted the data to test the correlation between the real permittivity and relaxation time T1. For the fitting function, we chose an equation that resembles the Debye Eq. (7.1). The fitting was performed using a nonlinear least square method, and the function used was

12 $$\varepsilon^{\prime} = e_{1} + \frac{{e_{2} - e_{1} }}{{1 + \left( {c T1} \right)^{2} }}$$

with coefficient values of *e*_1_ = 76.86, e_2_ = 37.71, and *c* = 0.0018, with T1 in milliseconds.

### Data analysis of loss index *ε’’* and T1

The fitting of the loss index with the T1 values does not show a strong correlation, as in the case of permittivity; however, in this study, we analyzed a couple of situations that could help to understand the correlation between both values. As described by Eq. (7.2), the loss index can be related to the relaxation time, given the permittivity at zero and high frequencies. In the case of the water phantom, applying this equation with the values of *ε*_*s*_ = 78, *ε*_*∞*_ = 5.2, and *w* = 0.0018, which are similar to those of water, yields a fit that includes the measured point in the curve, as illustrated in Fig. 4a. Fitting the rest of the measured points with this function would result in poor direct correlation. Nevertheless, some properties, such as the water and NaCl concentrations, can be used for classification. From the work reported in^28^, it is known that the loss index curve changes according to the water concentration; the curve changes in scale and displacement; therefore, for a specific frequency, the loss index value does not change linearly with the water concentration. Contrary to the permittivity, the value varies linearly with water content. A function that can be used for fitting these measured points would require scaling by the concentration of NaCl and displacement by an offset to T1. Although the Debye and Cole–Cole equations do not include a scaling factor for a given material, the mixing theory uses the concentration factor. The general Maxwell–Garnett mixture theory was applied in this study. We examine a function based on the Maxwell–Garnett mixing formula. For the case where the volume fraction represents NaCl, a fitting function similar to Eqs. (7.2) and (11) can be rewritten as

13 $$\varepsilon^{\prime\prime} = \frac{afT1}{{1 + \left( {bfT1} \right)^{2} }}$$

where *f* is the percentage of NaCl, which corresponds to the scaling factor.

Another equation that can be used to describe the loss index is the geometric representation of Eq. (8) between $\varepsilon^{\prime}$ and $\varepsilon^{\prime\prime}$. Based on Eq. (8) a relationship between the real permittivity and imaginary permittivity can be made including the scaling factor accounting for the percentage of NaCl as

14 $$\varepsilon^{\prime\prime} = \left( {\sqrt {k^{2} - \left( {\varepsilon^{\prime} - cr} \right)^{2} } } \right)sc$$

where *k* is the radius of the semicircle, *cr* is the center of the semicircle, and *sc* is a scaling factor that accounts for the increase in the dielectric loss because of the mixture with NaCl. The values of *cr* and *sc* can be determined by solving the boundary problem, in which *k*, *cr* and *sc* > 0, and maintaining the output of the real and positive functions.

In summary, the real and imaginary permittivity components of several samples were measured using a dielectric measurement kit at a frequency of 297.2 MHz corresponding to a 7 T MRI. T1 maps were acquired from the samples using the inversion recovery sequence and magnetization-prepared rapid gradient-echo (MPRAGE) pulse sequences. A rational equation similar to Debye equations was explored to fit the data between the measured dielectric data and T1 values. This study was performed using only phantoms, and the results can be verified by measuring the dielectric properties.

## Results

### Phantom development and dielectric measurement

The values of the measured electrical properties are presented in Table 1. The RMSE between the measured and the experimental values was 1.01. The values were collected at a frequency of 297.8 MHz, corresponding to the 7 T MRI scanner frequency of operation. Two sets of phantoms were used: one for IR imaging and the other for MPRAGE imaging. For IR imaging, we used gray matter properties as the background tissue for phantom 1 (Fig. 1a) and cerebellum (Fig. 1b) in phantom 2. For MPRAGE imaging, pure water was used as the background in phantom 1 (Fig. 1a) and oil in phantom 2 (Fig. 1b). The materials were changed independent of the pulse sequence and were changed only to add more tissues for analysis.

### T1 mapping

**Figure 2:**
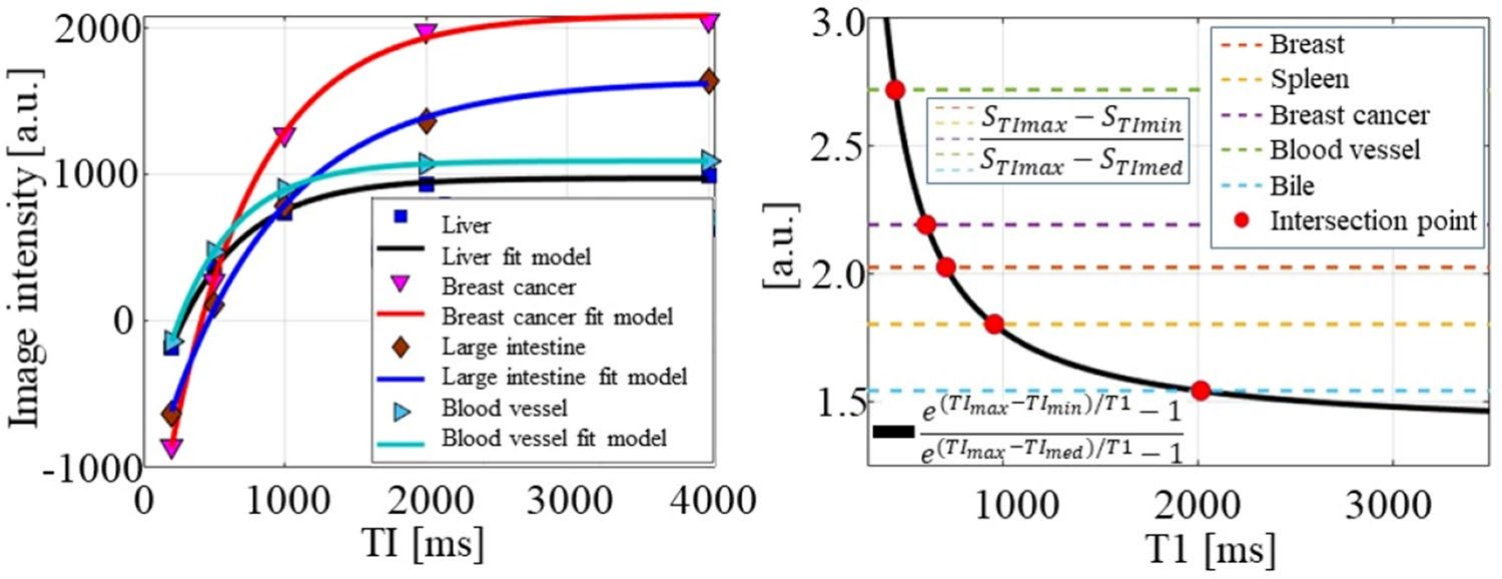
Plots of the computation of T1 by using (**a**) curve fitting for inversion recovery images. The plot (**b**) demonstrate the solution of Eq. (2), by finding the T1 value at which the look up table (black line) intersects the dotted lines corresponding to the MPRAGE images intensity of each phantom.

**Figure 3:**
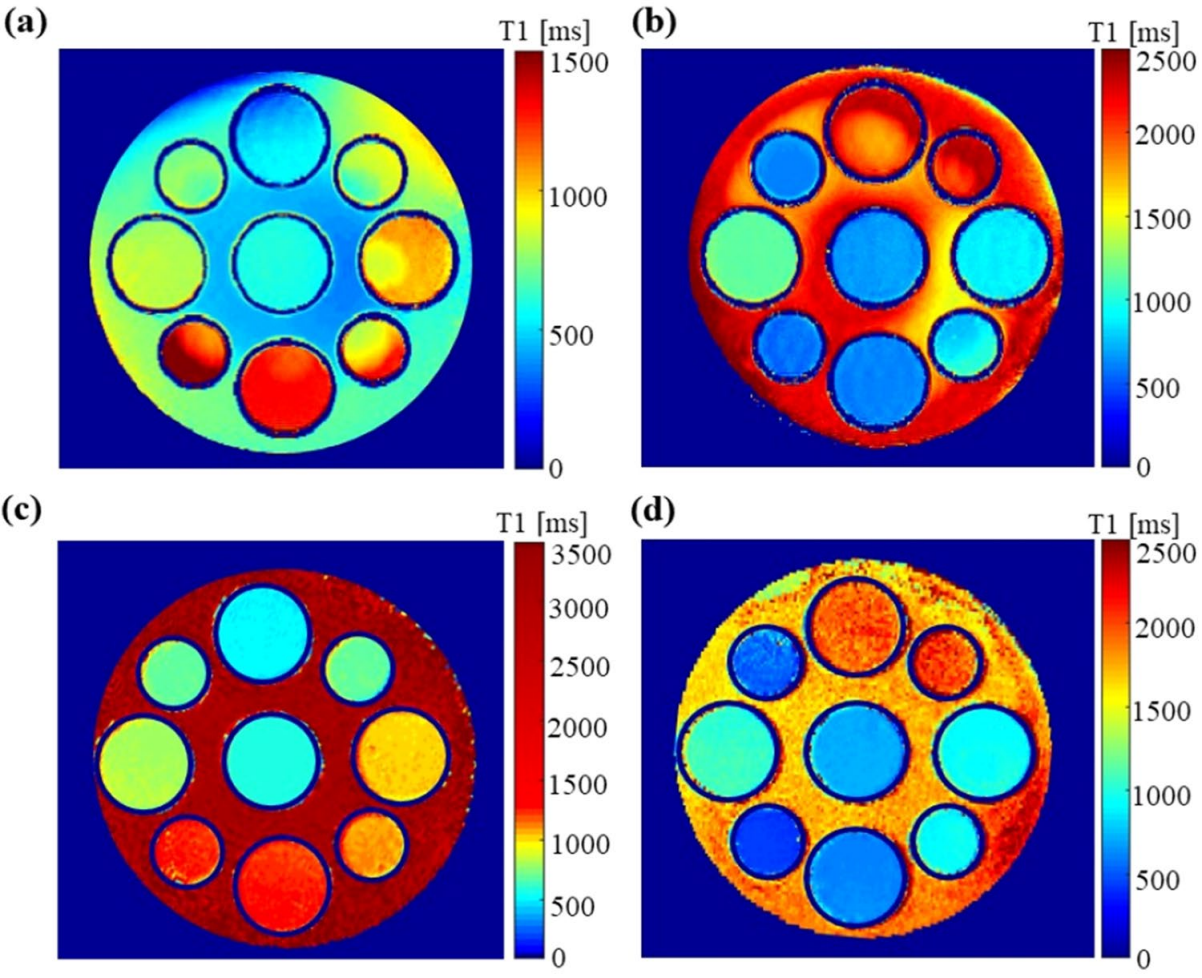
T1 maps acquired through the inversion recovery method for (**a**) phantom 1, with background of grey matter and (**b**) phantom 2 with the cerebellum compartment. The T1 maps acquired with MRPAGE for (**c**) phantom 1, with background of water, and (**d**) phantom 2, with oil phantom. The image shows the different T1 values for the individual phantoms mimicking tissues with specific electrical properties.

T1 values were computed using Eq. (1) for images acquired with inversion recovery. Figure 2a demonstrates the fitting model between image intensity and TI for the case of liver, breast cancer, large intestine, and blood vessel phantoms. T1, with the images acquired with MPRAGE, was computed using Eq. (2). The plot in Fig. 2b helps to visualize the method for solving Eq. (2), by plotting the right and left part and search the intersection. The right part of Eq. (2) is plotted as a black line, while the left part as a horizontal dotted line for the case of the breast, spleen, breast cancer, blood vessel and bile phantoms. The red circle in the figure illustrates the solution or intersection point between the left and right part of the equation, which corresponds to the T1 value of the respective phantom. The computed T1 maps for each phantom are shown in Fig. 3a,b. Because the use of the inversion recovery sequence is susceptible to B1-field nonuniformities, we also used MPRAGE T1 mapping by acquiring three MPRAGE images for each phantom with different TI values.

The T1 maps with MPRAGE are illustrated in Fig. 3c,d, and the estimated T1 values for each tissue type are presented in Table 2. T1 maps using MPRAGE resulted in more uniform values across each sample, especially in the background area.

**Table 2:** The computed T1 values for each of the tissue-mimicking samples.

Tissue type	Mean [ms]	STD [ms]	T1 difference
Gray matter (brain)	588	6.94	–
Muscle	568	1.94	6
Prostate	700	0.90	11
Stomach	1019	2.69	8.5
Gallbladder	696	4.42	10
Heart	1092	5.77	15
Kidney	1243	6.46	7.5
Large intestine	813	2.51	2.5
Small intestine	1247	9.22	24
Liver	496	0.68	4
CSF	1745	5.87	25
Bile	2033	20.97	15
Blood	946	5.23	6.5
Blood vessel	436	1.82	50
Breast gland	712	2.00	11.5
Spleen	936	5.86	14
Cerebellum	606	1.02	–
Cancer stomach	1984	3.71	8
Cancer colon	1073	6.09	14
Cancer breast	618	1.93	4
Water	3221	44.00	–
Oil	555	26.00	–

### Real permittivity and T1

**Figure 4:**
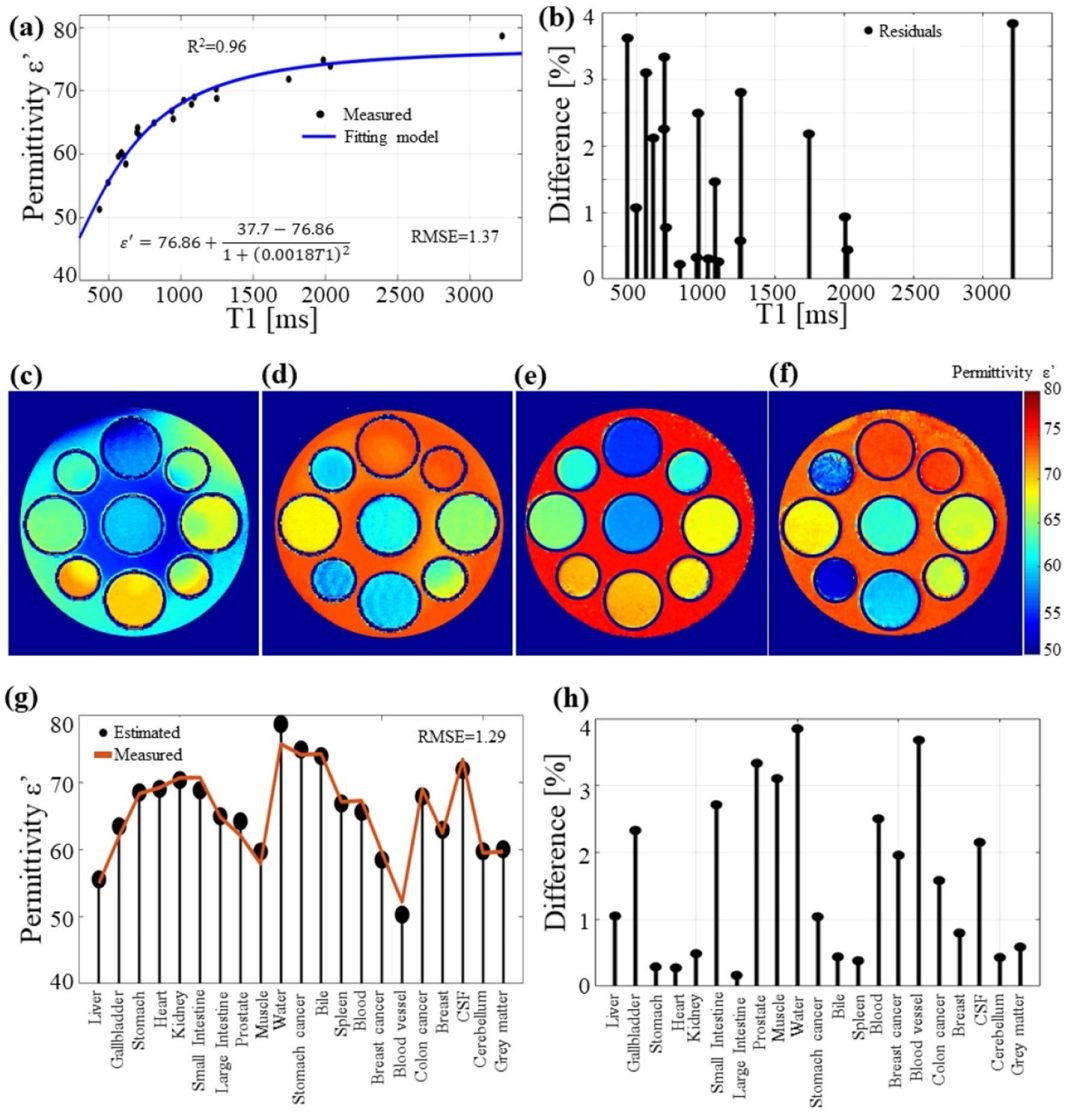
The curve fitting (**a**) between measured permittivity and T1. (**b**) The error in percentage of the measured points and the fitting function. The permittivity estimated using inversion recovery for (**c**) phantom 1 and (**d**) phantom 2 and using MPRAGE for (**e**) phantom 1 and (**f**) phantom 2. The statistical analysis between the measured values and the estimated permittivity (**g**), and the error in percentage between the estimated permittivity and the measured values (**h**).

The curve fitting using Eq. (12) and data samples for T1 and the measured permittivity of each phantom are shown in Fig. 4a and the difference between the estimated values with those of the fitting model is illustrated in Fig. 4b. The fitting had R^2^ and RMSE values of 0.96 and 1.37, respectively. From the plot, it can be observed that the samples fit the proposed equation. The only sample that did not fall into the fitting model was the oil phantom. The T1 of the oil phantom was computed to be 430 ms, while the permittivity was measured to be 11; when included in the fitting equation, the oil phantom had an error of 40. This was expected, as the rest of the samples were based on water.

From the fitting equation (Eq. 12), the values for the coefficients in the numerator correspond to the permittivity of the sample based on water. As illustrated in previous work^25,28^, at 300 MHz, the permittivity of the sample with 100% water is approximately 78, and at a low concentration of water, it is approximately 35. The value of c is a scaled value of the angular frequency w = 2*pi*297e^6^ = 1.8e^9^, with a scale of 1e^12^.

The permittivity maps computed using the curve fitting are illustrated in Fig. 4c–f, for the images obtained using IR in Fig. 4c and d and those obtained using MPRAGE in Fig. 4e and f for phantoms 1 and 2, respectively. A comparison between the estimated permittivity for each phantom and the measured data is illustrated in Fig. 4g. The difference between the estimated and measured data is illustrated in Fig. 4h for each phantom. The RMSE was 1.29. The results show a strong correlation between the T1 and the proposed fitting equation.

### Loss index *ε’’* and T1

**Figure 5:**
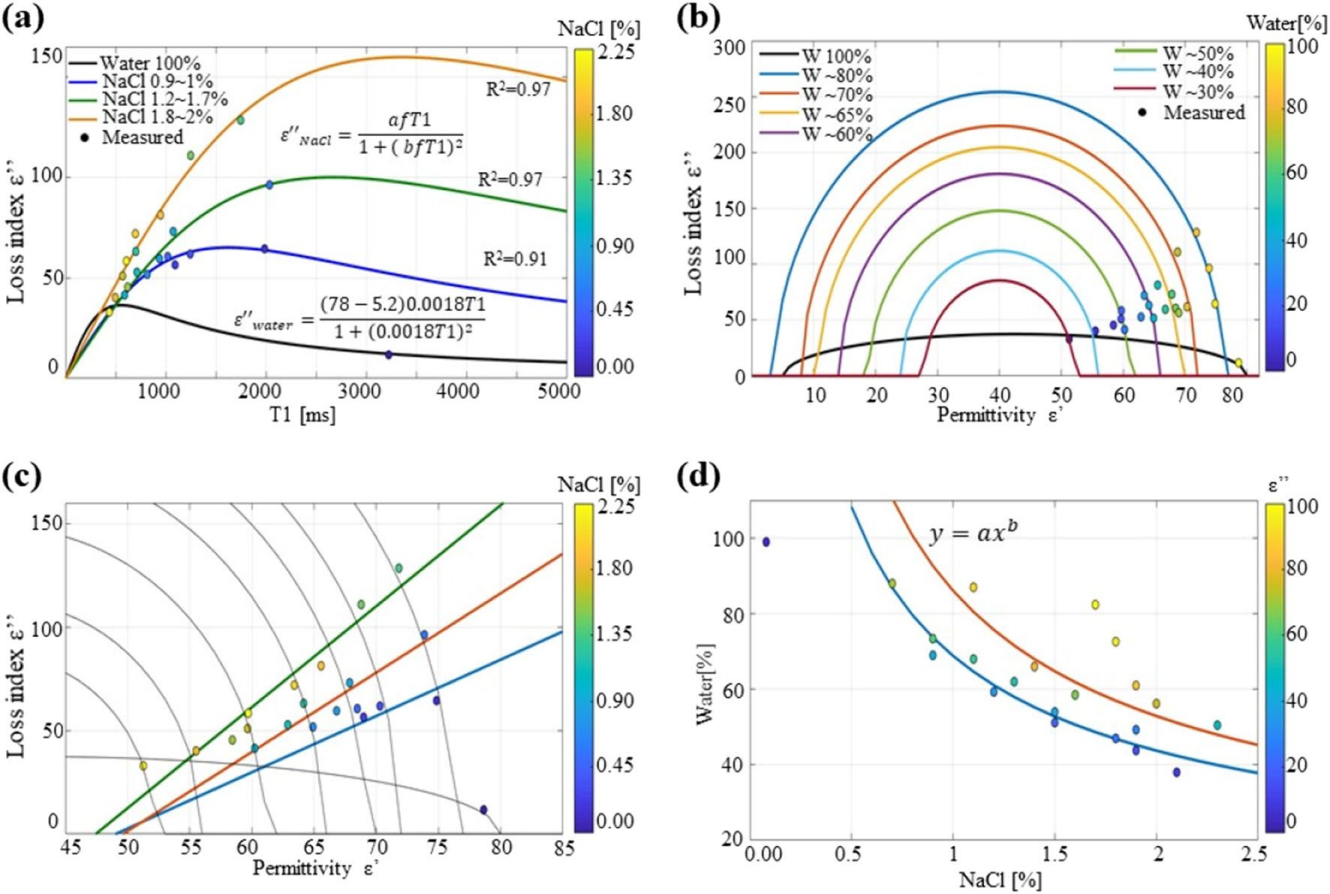
The curve fitting between T1 and the loss index (**a**) with the color curves indicating the concentration of NaCl. The plots of the permittivity and loss index, (**b**) with the color scale showing the concentration of water for each sample, and (**c**) with the color scale based on NaCl. The relationship between NaCl and water to the value of the loss index (**d**) as indicated by the dots in a color scale.

Using the fitting Eq. (13) the measured points can be fitted as illustrated in Fig. 5a. In this figure the measured loss index is plotted against T1 values for each phantom. Each point had a color value indicating the percentage of NaCl, and the fitting curves were classified into three sets. The first set consisted of 0.7% to 1.1% NaCl; when the factor *f* was set to 0.01, the variables were *a* = 7.82 and *b* = 0.06. The fitting curve is plotted in dark blue in Fig. 5a. This fitting had R^2^ = 0.91. The second set consisted of samples with NaCl concentration between 1.2 and 1.7%. For the fitting, *f* was assigned to 0.015, for which the variables were found to be *a* = 5.05 and b = 0.025, with R^2^ = 0.97. The curve is plotted in green in Fig. 5a. The last set, consisting of the highest NaCl concentrations between 1.8 and 2.2%, for which *f* was set as 0.02 and the variables *a* = 4.77 and *b* = 0.01492, yielded a fitting with R^2^ = 0.97. The curve is plotted in orange in Fig. 5a. The relationship between variables *a* and *b* as a function of *f* can be approximated as follows:

15.1 $$a = - 305f + 10.44$$

15.2 $$b = - 4.53f + 0.10$$

These equations are linear approximations, which had R^2^ of 0.80 and 0.90 for *a* and *b*, respectively.

The relationship between real and imaginary permittivity was explored by using Eq. (14). For the case of the water phantom, Eq. (14) is reduced to Eq. (8), which shows that the measured point lies inside the curve, as illustrated in Fig. 5b in the black curve. For the rest of the points, we select the fitting function of Eq. (14). The values of *cr* and *sc* were found to be 40 and 7, respectively, the value of *k* changed linearly with the percentage of water, and the curves are plotted in Fig. 5b. In this plot, we also used a color scale to display the water percentage of each of the samples measured. The value of *k* in relation to the water content was found to be linear, as follows:

16 $$k = 41.7W - 1.48$$

where *W* is the percentage of water in the sample. The results obtained using this function have similar behavior to those from previously reported curves with different concentrations of water^28^, which shows that a lower concentration of water would result in smaller curves. It also appears that the positions of the points inside the curve are related to NaCl concentration. We examine the plots of ε’ and ε’’ again. Here, the color of the points represents the NaCl percentage, as illustrated in Fig. 5c. A linear pattern can be observed in which the NaCl concentration increases the slope within the semicircles of the water concentration. The plot in Fig. 5d depicts the water versus NaCl percentage with ε” values in the color map. A power function can be used to approximate the measured values. This relationship is expected because the algorithm (recipe) for computing the components of the phantoms is also a power function, which uses water, sugar, and NaCl as variables.

**Figure 6:**
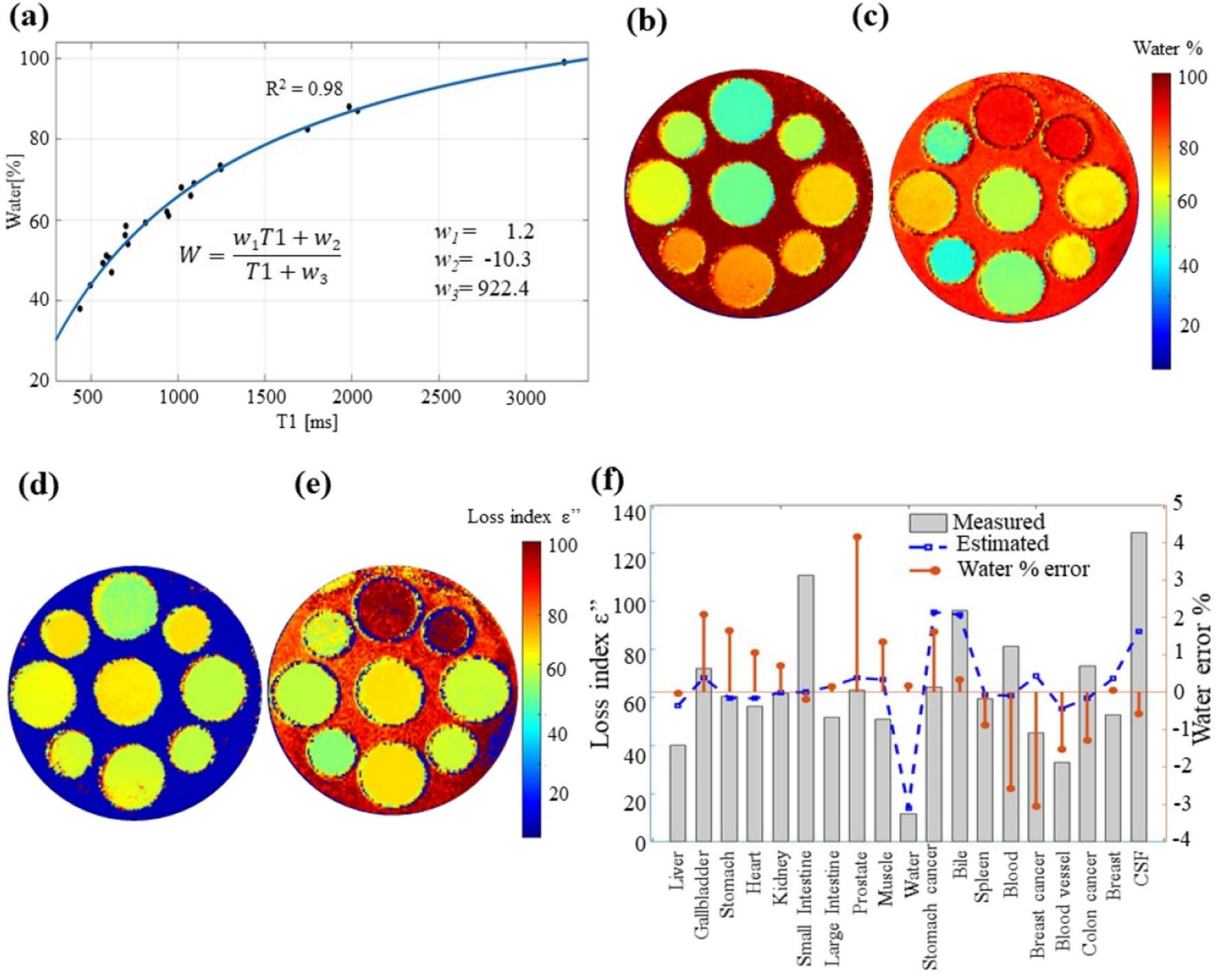
The curve fitting between T1 and water concentration (**a**). The water maps estimated based on T1 (**b**) phantom 1 and (**c**) phantom 2. The loss index estimated using the curve fit between permittivity and water for phantom 1, (**d**) and (**e**) 2. The comparison between the measured data and estimated loss index (**f**) including the water estimation error.

The previous analysis suggests that the sodium (Na) concentration should be known; however, this information can only be obtained by performing Na imaging, which requires a dedicated coil and hardware. Therefore, we tested only the fit between ε’ and ε’’. The fit between water and T1 is illustrated in Fig. 6a. By using a rational function of the first order, R^2^ of 0.98 was acquired. Applying the model to the T1 maps, water content maps were acquired, as illustrated in Fig. 6b,c. The ε’’ maps illustrated in Fig. 6d,e were computed using the functions in Eqs. (14) and (16) with the estimated water maps and ε’ maps. The comparison of the estimations is illustrated in Fig. 6f; the measured data are displayed in gray bars, the estimated ε’’ is indicated by the blue dotted line. The estimation of ε’’ has an RMSE value of 20. The water percentage error is also included in the plot in Fig. 6f, shown by the red line and right y-axis.

## Discussion

In this work, we explored the relationship between relaxation time T1 and complex permittivity. The T1 values acquired were based on the phantoms and did not represent real-human tissue T1 values. In the case of permittivity, this analysis showed a strong correlation between T1 and permittivity. The analysis demonstrated that a rational function similar to the Debye equation can be used to estimate the permittivity using T1. In particular, the coefficients obtained for the fitting function in (12) are similar to those used in Debye equations for water at a frequency of 300 MHz. The strong correlation between T1 and permittivity using Eq. (12) is expected to hold because the test phantoms were based on water, with a range of water percentages between 40 and 100%. However, the analysis of pure oil does not fall into the fitting function, with oil having a lower permittivity and conductivity than water. Therefore, a dedicated study should be performed to analyze the behavior of oil and its electrical properties. This future research could help us understand the relationship between fat tissues and their electrical properties.

In the case of the loss index or imaginary permittivity, the correlation between T1 is not as direct as in the case of permittivity. Using the Debye equation with the water parameters can accurately fit the point of the pure water phantom. However, with the addition of NaCl, the loss index of the other phantoms increased proportionally with the Na content. Therefore, using the concept of the mixing theory, Eq. (13) is scaled with the Na fraction. The proposed function is in accordance with the previous wEPT theory, where the conductivity and T1 show a direct correlation and can be expressed using the function in Eq. (13). These analyses suggest that the Na concentration should be known and can be acquired using Na imaging. The geometric correlation between the permittivity and index loss was also estimated using the relationship described by the Cole–Cole complex representation of permittivity. This analysis also reveals how semicircles formed between the index loss and permittivity can be used to estimate the index loss.

The current work was performed with a 7 T MRI system; however, similar results can be expected for other magnetic field systems. The previous work on w-EPT^14^ performed with a 3 T MRI system suggests the existence of such a correlation between T1 and electrical properties. Using the proposed Eq. (12) with the values of the table provided by the phantom study in^14^, the permittivity can be estimated with an R^2^ of 0.99; combining Eqs. (13) and (6), the conductivity with an R^2^ of 0.91. In this work, we presented a more general formulation of the model by using directly the T1 value not based on water. It should also be noted that the T1 values change according to the field strength^34,35^, as do the electrical properties based on the respective Larmor frequency^6^. In future, this assumption will be verified, and the current work is limited to the accessibility to other MRI systems.

In summary, the results of this work have demonstrated a strong correlation between T1 relaxation and the real part of permittivity. A good estimation of the permittivity of water-based mixtures can be obtained using the proposed fitting model based on Debye’s equations. The analysis of the imaginary part of the permittivity also indicates that can be estimated based on the relaxation and mixture theory. We hope that this study can help us have a better understanding of the relationship of mixture elements, relaxation times, and electrical properties. The analysis in this work can be also extended to elements other than sodium and sucrose, such that their effects on the electrical properties can be better understood.

## Data Availability

The datasets used and/or analyzed during the current study are available from the corresponding author upon reasonable request.
